# Expression of TopBP1 in hereditary breast cancer

**DOI:** 10.1007/s11033-012-1622-z

**Published:** 2012-04-28

**Authors:** Ewa Forma, Anna Krzeslak, Magdalena Bernaciak, Hanna Romanowicz-Makowska, Magdalena Brys

**Affiliations:** 1 Department of Cytobiochemistry, University of Lodz, Pomorska 141/143, 90-236 Lodz, Poland; 2 Department of Clinical Pathomorphology, Polish Mother’s Memorial Hospital Research Institute, Rzgowska 281/289, 93-338 Lodz, Poland

**Keywords:** TopBP1, Gene, Protein, Expression profiling, Hereditary breast cancer

## Abstract

TopBP1 protein displays structural as well as functional similarities to BRCA1 and is involved in DNA replication, DNA damage checkpoint response and transcriptional regulation. Aberrant expression of TopBP1 may lead to genomic instability and can have pathological consequences. In this study we aimed to investigate expression of *TopBP1* gene at mRNA and protein level in hereditary breast cancer. Real-time quantitative PCR was performed in 127 breast cancer samples. Expression of *TopBP1* mRNA in lobular carcinoma was significantly lower compared with ductal carcinoma (*p* < 0.05). The level of *TopBP1* mRNA appeared to be lower in poorly differentiated (III grade) hereditary breast cancer in comparison with moderately (II grade) and well-differentiated cancer (I grade) (*p* < 0.05 and *p* < 0.001 respectively). We analyzed TopBP1 protein expression using immunohistochemistry and Western blot techniques. Expression of TopBP1 protein was found to be significantly increased in poorly differentiated breast cancer (III grade) (*p* < 0.05). The percentage of samples with cytoplasmic apart from nuclear staining increased with increasing histological grade. There was no significant association between level and intracellular localization of TopBP1 protein in hereditary breast cancer and other clinicopathological parameters such as estrogen and progesterone receptors status, appearance of metastasis in the axillary lymph nodes and type of cancer. Our data suggest that decreased level of *TopBP1* mRNA and increased level of TopBP1 protein might be associated with progression of hereditary breast cancer.

## Introduction

TopBP1 protein (topoisomerase IIβ binding protein 1) was identified as an interacting partner for topoisomerase IIβ [1, 2]. TopBP1 possesses eight BRCA1 (breast cancer gene 1) carboxyl-terminal (BRCT) domains which were for the first time described for BRCA1 protein and are commonly found in proteins involved in regulation of the cell cycle checkpoint and the response of the cell to DNA damage [1, 3–5]. The C-terminal region of TopBP1 containing two BRCTs shows considerable similarity to the corresponding part of BRCA1. Beside the sequence homology, TopBP1 also displays functional similarities to BRCA1 [6, 7]. Both proteins are strongly induced during S phase. Following ionizing radiation, TopBP1 is recruited to DNA breaks and colocalizes with NBS1 (Nijmegen breakage syndrome protein 1), BRCA1, and 53BP1 (p53 binding protein 1) in nuclear foci [7–9]. TopBP1 interacts with several other proteins, including human papilliomavirus type 16 (HPV16) transcription/replication factor E2, DNA polymerase ε, checkpoint protein hRad9, and transcription factor E2F1 [8, 10–15]. Thus, TopBP1 seems to be involved in DNA replication, DNA damage checkpoint response and transcriptional regulation [3, 9, 13, 16–19]. TopBP1 protein is essential for maintenance of chromosomal integrity and cell proliferation. This protein appeared to be involved in DNA damage response, DNA replication checkpoint, chromosome replication and regulation of transcription [20–22]. TopBP1 knockout mouse exhibits early embryonic lethality at the peri-implantation stage and TopBP1 deficiency induces cellular senescence in primary cells [20, 22].

Genomic stability in eukaryotic cells is maintained by checkpoint mechanisms which coordinate cell cycle progression and other processes including transcription, apoptosis and DNA repair [3]. Some signals of DNA damage can lead to cell cycle arrest to prevent transfer of damaged genetic information to the daughter cells. Checkpoint responses are considered to be a major mechanism to reduce both initiation and progression of cancer, which can be caused by incomplete DNA repair, resulting in genetic alterations of tumor suppressor genes and protooncogenes [7]. TopBP1 appears to be directly involved in controlling replication initiation. In this regard, its role in repressing E2F1-mediated apoptosis at the G1/S transition would be crucial to ensure suppression of apoptosis before initiation of DNA replication. Like other proteins that are directly involved in DNA replication, TopBP1 is induced by E2F1 during G1/S transition. Therefore, TopBP1 acts as a critical cooperator to enforce the execution of S phase [3, 14, 17]. Both, TopBP1 and BRCA1 specifically regulate the G2/M checkpoint partially compensating each function [7]. At G2/M checkpoint, TopBP1 binds and activates ATR (ATM and Rad3-related protein) in an ATRIP (ATR interacting protein)-dependent manner, leading to recruitment and phosphorylation of BRCA1 and subsequent activation of Chk1 (checkpoint kinase 1) and other ATR effectors [23, 24]. Activation of Chk1 can lead to cell cycle arrest and damage repair [7, 14].

Aberrant expression of TopBP1 may be involved in the deregulation of processes controlled by this protein and can have pathological consequences. Therefore, the current study was aimed at investigating expression of *TopBP1* gene at mRNA and protein level in hereditary breast cancers.

## Materials and methods

### Specimen collection

The studied material was obtained from Polish Mother’s Memorial Hospital, Research Institute, Lodz, Poland and comprised of 127 of formalin-fixed paraffin embedded (FFPE) sections of hereditary breast cancers (patients age range 28–69 years) and 24 normal breast tissues (patients age range 35–59 years). Apart from FFPE for 50 of 127 breast cancer patients fresh frozen (−80 °C) breast tissues were also obtained. Blood samples were obtained for each patient. Normal tissues were obtained from women undergoing radical mastectomy and after resection were immediately frozen at −80 °C. Cancerous and normal tissues belong to different persons.

Criteria for classifying the samples as hereditary breast cancers were as follows (1) at least one first-degree relative with breast cancer, regardless of age (*n* = 92), (2) breast cancer diagnosed below 40 years of age (*n* = 35). We analyzed three *BRCA1* mutations (C61G, 4153delA, 5382insC) in DNA of studied breast cancer patients (see details in Method section). BRCA1 mutations were detected in 72 of 127 of studied patients. None of the patients received neoadjuvant endocrine therapy, chemotherapy or radiotherapy. A structured questionnaire was used to collect detailed information on age, age of menarche and/or menopause, weight, and family history of cancer. The pathological evaluation report was obtained for each patient (Table 1).

**Table 1:** Characteristics of patient and tumor samples

Characteristics	Patients	
	*N*	%
Normal breast tissue	24	
Age range	35–59	
Mean ± SD	48.5 ± 10.5	
Breast cancer	127	
Age of diagnosis		
Range	28–69	
Mean ± SD	54.3 ± 11.5	
Type of cancer		
Ductal carcinoma	99	78
Lobular carcinoma	28	22
Tumor grade according to Bloom–Richardson system		
I	14	11
II	76	60
III	37	29
Lymph node metastasis		
No	72	56
Yes	55	44
Menopausal status		
Premenopausal	73	57
Postmenopausal	54	43
ER status		
Negative	61	48
Positive	66	52
PR status		
Negative	68	33
Positive	59	67

*ER* estrogen receptor, *PR* progesterone receptor

### Analysis of *BRCA1* mutations

Mutations of *BRCA1* and *BRCA2* genes confer a high lifetime risk for both breast and ovarian cancer. Many different *BRCA1* and *BRCA2* mutations have been described in families with early-onset breast and ovarian cancer [25–28]. In Poland three mutations (C61G, 4163delA and 5382insC) in *BRCA1* gene account for 86 % of all *BRCA1* and *BRCA2* mutations [29, 30]. Mutations in *BRCA2* are relatively rare in Poland and no founder *BRCA2* mutations have been identified [29, 30]. Therefore, we analyzed presence of C61G, 4163delA and 5382insC in studied patients with breast cancer.

Genomic DNA was prepared from peripheral blood by using the commercial GenElute Blood Genomic DNA Kit (Sigma Aldrich, Germany) according to the manufacturer’s instruction. The allele specific oligonucleotides polymerase chain reaction (ASO-PCR) was used to determine the studied mutations of *BRCA1* gene. PCR assays were performed in a total reaction volume of 25 μl containing 50 ng of genomic DNA, 1U Taq DNA polymerase (Sigma Aldrich, Germany), 1× reaction buffer (10 mM Trizma HCl, pH 8.3; 50 mM KCl; 1.5 mM MgCl_2_; 0.001 % (w/v) gelatin), 200 μM of each dNTP, 0.25 μM of each primer. PCR amplifications were conducted in GeneAmp PCR System 9700 (Perkin-Elmer, USA). Thermal cycling conditions were as follows: initial denaturation step at 94 °C for 5 min, 30 cycles at 95 °C for 30 s, 30 s at 54 °C for C61 > G, 51 °C for 4153delA and 60 °C for 5382insC, and 60 s at 72 °C. The terminal extension step was performed for 10 min at 72 °C. The following allele specific oligonucleotides as primers were used: F:5′GGTTTCTCAGATAACTGGGCC3′ (wild type variant), F:5′GGTTTCTCAG-ATAACTGGGCG3′ (mutated type variant) and R:5′CGTCAAAGAATACCCATCTG3′ (common primer) for C61G mutation; F:5′GGCATCTCAGGAACATCACC3′ (common primer), R:5′CTTGCCCGTTCCTCTTTCTTC3′ (wild type variant) and F:5′CTTGCCCGTTCCTCTTTCTGA3′ (mutated type variant) for 4153delA mutation; F:5′TGTTGGTCAGACTGGTGTCG3′ (common primer), F:CATTGACCAC-ATCTCCTCTGAC3′ (wild type variant) and F:5′CATTGACCACATCTCCTCTGGA3′ (mutated type variant) for 5382insC mutation. The PCR products (163, 220 and 225 bp) were separated onto 8 % polyacrylamide gel, stained with ethidium bromide and viewed under UV light.

### Total RNA extraction from FFPE breast cancer sections and fresh frozen normal and cancerous breast tissues

Sections were deparaffinized by two rinses in xylene for 10 min at room temperature with shaking, followed by centrifugations at room temperature for 5 min at 12,000× *g*. After deparaffinization, we introduced a rehydratation step (rinsing in 100% ethanol, 85, 70 % ethanol, all prepared with diethylpyrocarbonate (DEPC) treated dH_2_O, for 5 min). The tissue was collected by centrifugation at 12,000× *g* for 5 min. After the final wash, alcohol was aspirated. The dried tissue pellets of breast cancer samples and fresh frozen normal and cancer breast tissues were resuspended in 500 μl of digestion buffer (10 mM NaCl; 500 mM Tris–HCl, pH 8.0; 25 mM EDTA; 1 % SDS) and 1 mg/ml proteinase K was added. Samples were incubated at 45 °C overnight. Prior to RNA purification, in same samples we inactivated proteinase K at 97 °C for 10 min. The digested samples were extracted using TRI Reagent (Sigma Aldrich, USA) according to manufacturer’s protocol. RNA was eluted in 20 μl RNase-free water, quantified by spectrophotometry at 260 nm and stored at −20 °C. RNA with a 260/280 nm ratio in range 1.8–2.0 was considered high quality. Integrity was evaluated by assessing the 18S and 28S rRNA bands in 1 % ethidium bromide stained agarose gels.

### cDNA synthesis

First strand cDNA was synthesized from each RNA pool using PCR Kit ver 3.0 (Takara Bio Inc., Japan) according to the manufacturer’s instruction. Briefly, 1 μg RNA was combined with 2.5 pmol of oligo dT-adapter primer, 4 μl of 25 mM MgCl_2_, 2 μl 10× RNA PCR buffer, 2 μl of 10 mM dNTP mixture, 20 units of RNase inhibitor, 5 units of AMV reverse transcriptase XL, and RNase-free water to total volume of 20 μl. The reaction took place at 42 °C for 30 min, followed by 95 °C for 5 min and 5 °C for 5 min in a GeneAmp PCR System 9700 (Perkin-Elmer Co, USA). cDNA was stored at −20 °C.

### Real-time quantitative PCR

The real-time PCR was performed in Mastercycler ep Realplex 4S (Eppendorf, Germany). Primers and TaqMan probes for *TopBP1* and *GAPDH* control reference gene were designed and synthesized according to TaqMan Gene Expression Assay (assay Hs00199775_m1 and Hs00266705_g1, respectively) (Applied Biosystems, USA). PCR reactions were carried out in a total volume of 10 μl of universal master mix (Applied Biosystems, USA) and 1 μl cDNA. The reactions were performed in duplicate. A positive result was defined by a threshold cycle (Ct) value lower than 40 (the Ct value is determined by the number of cycles needed to exceed the background signal). Ct value of all positive results were lower than 30. Abundance of *TopBP1* mRNA in samples was quantified by the ΔCt method. ΔCt (Ct_TopBP1_ − Ct_GAPDH_) values were recalculated into relative copy number values (number of copies of *TopBP1* mRNA per 1,000 copies of *GAPDH* mRNA).

### Sequencing of PCR products

We amplified fragment of *TopBP1* cDNA by PCR and purified products using QIAquick purification columns (Qiagen, USA). Both strands were sequenced using a BigDye Terminator Cycle Sequencing Kit ver. 1.1 (Applied Biosystems, USA) according to the manufacturer’s recommendations. Reactions were analysed on an ABI Prism 377 DNA Sequencer (Applied Biosystems, USA).

### Immunohistochemistry

2 μm tissue sections were deparaffinized and rehydrated through xylene and graded ethanol, respectively. Slides were rinsed in dH_2_O, then were subjected to antigen retrieval in 10 mM citrate buffer, pH 6.0 by microwave oven heating for 20 min. Endogenous peroxidase activity was quenched in 0.3 % H_2_O_2_ for 30 min, then washed in dH_2_O. After blocking in Ready-to-Used Blocking Reagent (Bethyl, USA), slides were incubated 60 min at room temperature with rabbit anti-human TopBP1 antibody (Abcam, ab2402), dilution 1:200. Detection was performed with Immunohistochemistry Accessory Kit IHC-101 (Bethyl, USA) according to the performed by incubating the sections in a solution of DAB. Sections were counterstained with hematoxylin, dehydrated and coverslipped. For each antibody and samples a negative control was processed.

### Evaluation of staining

Each case was evaluated in terms of staining intensity as positive and negative cells. The immunoreactive score (IRS) of the TopBP1 was estimated by multiplying the score for staining intensity (0—negative, 1—weak, 2—moderate and 3—strong staining) and score for the percentage of positively stained cells (0 = 0 %; 1 = <5 %; 2 = 5–35 %; 3 = >35–70 % and 4 = >70 %). An IRS score of 6 or higher was considered to be a strong reactivity, 4–5 moderate, 2–3 weak and 0–1 negative. The immunoreactive score results were estimated by two pathologists.

### Western blot analysis

Cytoplasmic and nuclear fractions were separated from 50 cancerous and 24 normal fresh frozen breast tissue samples by differential centrifugation of tissue homogenate in isotonic sucrose in the presence of the serine protease inhibitor PMSF and 10 mM sodium molybdate. Nuclei were finally purified by centrifugation through 2.2 M sucrose. Protein concentration in homogenates and cellular fractions was evaluated according to Lowry protocol using bovine serum albumin (BSA) as standard. Proteins of homogenate as well as cytoplasmic and nuclear fractions (50 μg) were resolved on 8 % SDS-PAGE and electrotransferred onto Immobilon-P membrane (Millipore, USA). The loading and efficiency of transfer were verified by Ponceau S staining of membranes. After blocking in 0.5 % casein the membrane was incubated with specific primary anti-TopBP1 antibody diluted (1:5,000) in TBS with 0.5 % casein at 4 °C for 12 h. Commercial polyclonal antibody specific for the portion of human TopBP1 encoded within exon 28 was used (Abcam, UK). Following extensive washing with TBST buffer (Tris-buffered saline with Tween 20) the membrane was incubated with mouse anti-rabbit IgG-HRP antibody (Santa Cruz, USA). The specificity of antigen–antibody interaction was visualized with 4-chloro-1-naphthol and hydrogen peroxide as substrate for horseradish peroxidase (HRP). The intensities of the visualized signals were analyzed densitometrically.

### Statistical analysis

Statistical analysis was performed using the STATISTICA version 9.0 (SatatSoft, Poland). Analysis of differences between *TopBP1* mRNA and clinical parameters was carried out using the Mann–Whitney test and Kruskal–Wallis test with post hoc multiple comparisons. Relationships between TopBP1 protein expression and intracellular localization and clinicopathological factors were examined with Chi^2^ test. Analysis of the relationship between mRNA and protein level was performed using Spearman rank test. *p* value of *p* < 0.05 was considered statistically significant.

## Results

**Fig. 1:**
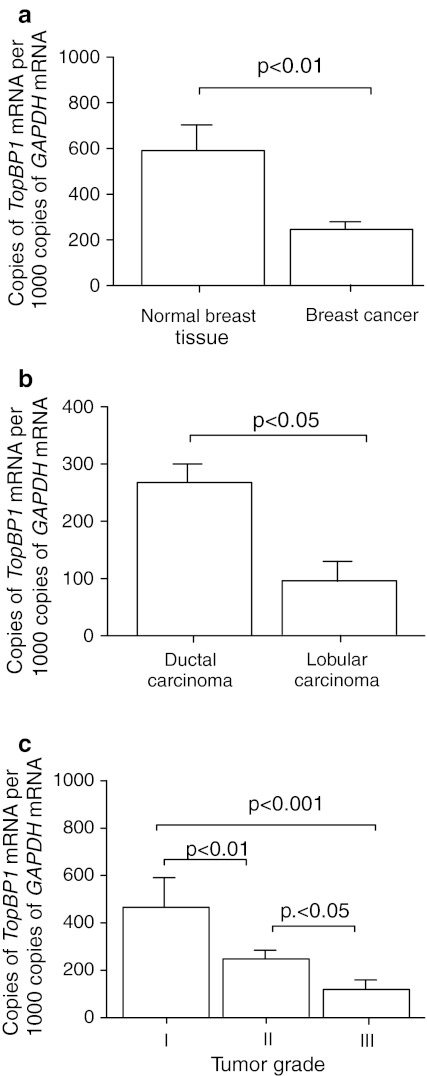
Expression of *TopBP1* mRNA measured by real-time PCR. **a** in normal breast tissue and hereditary breast cancer, **b** in relation to type of cancer and **c** tumor grade. Graphs represent mean ± SEM

The expression of *TopBP1* gene at the mRNA level in normal breast tissue and hereditary breast cancer was estimated by real-time quantitative PCR analysis with *GAPDH* gene applied as a reference. Sequencing of PCR products confirmed that amplification product correspond with full-length sequence of TopBP1 and there were no other isoforms. In case of 50 cancer samples *TopBP1* mRNA expression was analyzed both in FFPE sections and fresh breast tissues. Since there was no differences between mRNA expression in FFPE samples and fresh tissues only results from FFPE sections are presented on diagrams and table. The comparison of *TopBP1* mRNA level with different clinicopathological parameters of tumors is shown in Table 2, where the average of *TopBP1* mRNA copies per 1,000 copies of *GAPDH* mRNA represent the mean level of *TopBP1* mRNA, and are used for statistical analysis. *TopBP1* mRNA expression was observed in all of 24 (100 %) normal breast tissue samples and in 97 of 127 (76.4 %) breast cancer samples. Thus, positive expression of *TopBP1* mRNA in normal tissue was more frequently observed compare to breast cancer samples (*p* < 0.05). Level of *TopBP1* mRNA was significantly higher in normal breast tissues than in breast cancer specimens (*p* < 0.01; Fig. 1a). Expression of *TopBP1* mRNA was found to be significantly decreased in the lobular carcinoma compared to the ductal carcinoma (*p* < 0.05; Fig. 1b). Fifteen of 28 (53.6 %) lobular carcinoma demonstrated detectable mRNA for *TopBP1* gene, while the expression of *TopBP1* mRNA was observed in 76 of 99 (78.3 %) ductal carcinoma. Expression of *TopBP1* gene at the mRNA level was observed in 62.0 % (23/37) of poorly differentiated breast cancer (grade III), whereas nearly 80 % of tumors in I and II grade demonstrated detectable *TopBP1* mRNA (78.6 % (11/14) and 78.9 % (60/76), respectively). Significantly lower *TopBP1* mRNA level in the poorly differentiated (grade III) familial breast cancer compared with moderately (grade II, *p* < 0.01) and well-differentiated cancer (grade I, *p* < 0.001) was noted (Fig. 1c). There was no statistically significant difference in the context to clinicopathological parameters such as estrogen and progesterone receptor status and appearance of metastasis in the axillary lymph nodes.

**Table 2:** Expression of *TopBP1* mRNA in cancerous and normal breast tissues

Clinicopathological features (*N*)	Quantitative RT-PCR (copies of *TopBP1* mRNA per 1000 copies *GAPDH* mRNA)	*p*
Normal breast tissue (24)	587.2 ± 108.4	
Breast cancer (127)	243.0 ± 27.9	0.002
Type of cancer		
Ductal carcinoma (99)	266.9 ± 31.2	
Lobular carcinoma (28)	94.6 ± 34.1	0.03
Tumor grade		
I (14)	465.4 ± 121.6	
II (76)	245.1 ± 32.7	
III (37)	118.9 ± 34.4	0.003
Lymph node status		
No (72)	282.9 ± 40.8	
Yes (55)	196.8 ± 35.6	0.12
ER status		
Negative (61)	218.0 ± 37.9	
Positive (66)	269.8 ± 40.6	0.35
PR status		
Negative (68)	204.6 ± 38.3	
Positive (59)	276.7 ± 39.4	0.71

*ER* estrogen receptor, *PR* progesterone receptor

**Fig. 2:**
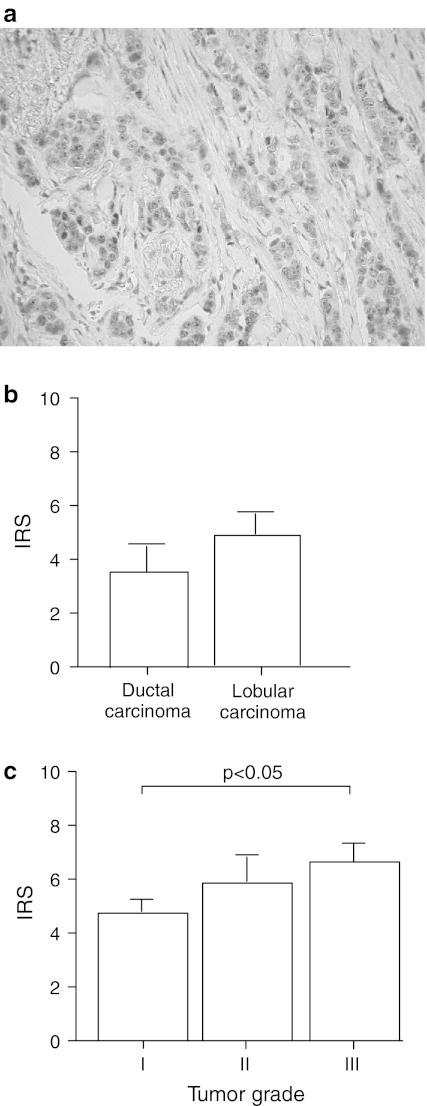
Immunohistochemical analysis of TopBP1 protein expression. **a** representative TopBP1 immunostaining results for formalin-fixed paraffin-embedded tissue of carcinoma ductale (original magnification, ×200), **b** expression of TopBP1 protein in relation to type of cancer and **c** tumor grade

The expression level of TopBP1 protein was determined in 127 breast carcinoma samples using immunohistochemical technique. The results concerning correlation between TopBP1 expression and clinicopathological parameters of breast cancers are shown in Fig. 2 and Table 3. TopBP1 was expressed in 121 of 127 (95.3 %) breast cancers. In 80/121 (66.1 %) there were heterogenous immunoreactivity with areas of nuclear and cytoplasmic staining (4 cases in grade I, 52 cases in grade II and 24 cases in grade III). Of the 121 cases, 51 (40.1 %) had a weak expression of TopBP1 protein (IRS score 2–3), 61 (48.0 %) a moderate expression (IRS score 4–5) and 9 (7.1 %) a strong expression (IRS score ≥6). Pathological grading was associated with TopBP1 protein expression. All tumor samples classified as grade I and grade II showed TopBP1 protein expression. In grade I, 12 cases had a weak and 2 a moderate expression and 0 cases strong TopBP1 protein expression. Six of 37 samples characterized as grade III had a negative TopBP1 protein expression, whereas 4 had a weak, 22 a moderate, and 5 a strong TopBP1 protein expression. There was no expression difference between type of tumor, lymph node status, and steroid hormone receptors status (Fig. 2; Table 3).

**Table 3:** Correlation between TopBP1 protein expression and clinicopathological findings

Clinicopathological features (*N*)	IRS No (%)				*p*
	Negative 0–1	Weak 2–3	Moderate 4–5	Strong ≥6	
Hereditary breast cancer (127)	6 (4.7)	51 (40.1)	61 (48.0)	9 (7.1)	
Type of cancer					
Ductal carcinoma (99)	6 (6.1)	36 (36.4)	49 (49.5)	8 (8.0)	0.25
Lobular carcinoma (28)	0 (0)	15 (53.6)	12 (42.8)	1 (3.6)	
Tumor grade					
I (14)	0 (0)	12 (85.7)	2 (14.3)	0 (0)	<0.0001
II (76)	0 (0)	53 (69.7)	20 (26.3)	3 (3.9)	
III (37)	6 (16.2)	4 (10.8)	22 (59.4)	5 (13.5)	
Lymph node status					
No (72)	4 (5.6)	32 (44.4)	27 (37.5)	4 (5.5)	0.91
Yes (55)	2 (3.6)	30 (54.5)	24 (43.6)	4 (7.3)	
ER status					
Negative (61)	2 (3.3)	35 (57.4)	30 (49.2)	3 (4.9)	0.81
Positive (66)	4 (6.1)	27 (40.9)	21 (31.8)	5 (7.6)	
PR status					
Negative (68)	3 (4.4)	34 (50.0)	27 (39.7)	4 (5.9)	0.99
Positive (59)	3 (5.1)	28 (47.4)	24 (40.7)	4 (6.8)	

*ER* estrogen receptor, *PR* progesterone receptor

**Fig. 3:**
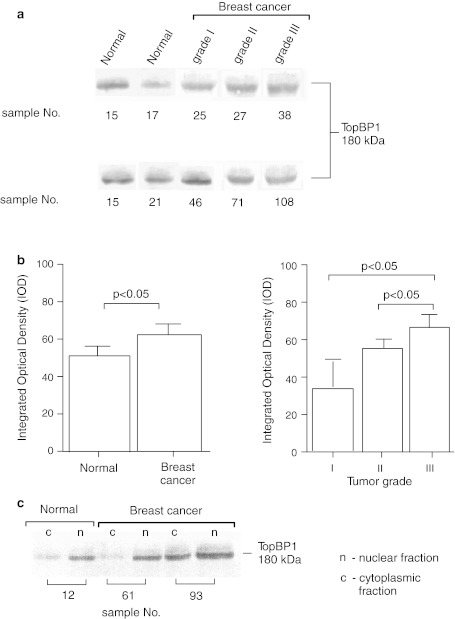
Western blot analysis of TopBP1 protein expression and localization. **a** TopBP1 protein expression in homogenate samples of normal and cancer breast tissue (sample no. 15 was used as a reference sample), **b** results of densitometric analysis of the intensity of TopBP1 bands in normal and cancer breast samples, and in relation to tumor grade, **c** nuclear and cytoplasmic localization of TopBP1 in representative normal and breast cancer tissues

Expression of TopBP1 protein was also examined using Western blot, in both normal breast tissue and carcinoma specimens in 24 and 50 cases, respectively. The results concerning TopBP1 expression in homogenates and its cellular localization are shown in Fig. 3c. Among 50 analyzed tumors, 4 were classified as grade I, 30 as grade II and 16 as grade III. The mean level of TopBP1 in homogenates was higher in cancer samples compared to normal cells (*p* < 0.05). Moreover, we observed higher TopBP1 protein level in the poorly differentiated breast cancers compared to moderately and well-differentiated cancers (Fig. 3a, b). These results are consisted with the results obtained with immunohistochemistry method (Fig. 2).

TopBP1 protein was found in the nuclear fraction of all normal breast tissue samples. Low level of TopBP1 was also detected in cytoplasmic fraction of seven normal tissue specimens. TopBP1 protein was found in all nuclear and one cytoplasmic fraction of analyzed breast cancer in grade I. Pattern of TopBP1 protein expression in well-differentiated breast cancer was similar to normal breast tissue samples. In 10 of 30 moderate differentiated breast cancers expression of TopBP1 was observed only in the nuclear fraction. In the remaining 20 tumor samples in grade II, TopBP1 protein was also detected in cytoplasmic fraction. However, level of this protein was lower in cytoplasmic fraction than nuclear fraction. In the case of four poorly differentiated breast cancer, TopBP1 protein was in the nuclear fraction and was absent in the cytoplasmic fraction. In 6 of 16 breast cancer in grade III, TopBP1 protein was reveals in both the nuclear and cytoplasmic fractions. However, the level of TopBP1 protein in two of these samples was higher in cytoplasmic fraction than in the nuclear fraction.

The results of TopBP1 protein expression showing higher TopBP1 level in cancer samples compared to normal tissues are in contrast to the results concerning mRNA level of *TopBP1*. In most cases of tumors we observed lower level of mRNA level than in normal tissue. Moreover, in most cases of tumors we found an inverse correlation between protein and mRNA level (Spearman’s rank analysis, *R* = −0.61, *p* > 0.05).

## Discussion

Breast cancer is the commonest malignancy in women and it is estimated that a million women worldwide will develop breast cancer each year [31]. About 5–10 % of the cases are considered familial [6]. A number of high penetrance breast cancer susceptibility genes have been identified and include *BRCA1* and *BRCA2*. These genes confer a high risk of breast and ovarian carcinoma. About 40–50 % of familial breast cancer can currently be explained by mutations in *BRCA1* and *BRCA2* genes. Of the remaining cases no more than 5 % are caused by defects in other studied genes, such as *p53*, *PTEN*, *ATM* and *Chk2* [6, 32, 33]. It is not known how many more genes that confer a small risk are yet to be identified or how these genes come together or interact with each other or with environmental factors to increase to breast cancer risk [31]. Most of the known cancer susceptibility genes encode proteins involved in the monitoring of genome integrity. Therefore, Karppinen et al. [6] suggested, that *TopBP1* coding for protein displaying structural and functional similarities with BRCA1 can be a plausible susceptibility gene for hereditary breast and ovarian cancer.

In this study we examined the relationship between expression of *TopBP1* gene at the mRNA and protein level and clinicopathological parameters of hereditary breast cancers. In the literature there is no data concerning expression of *TopBP1* at mRNA level in normal and cancerous tissues and only a few studies have examined the expression of TopBP1 protein in female breast cancer [34–37].

Our results obtained by immunohistochemical and Western blot analyses showed higher level of TopBP1 protein in breast cancer samples compared with normal breast tissues. Moreover, patients with overexpression of TopBP1 tend to have higher grades of breast cancer than those without overexpression of TopBP1. These results are consistent with results of Liu et al. [37] who have also showed higher expression of TopBP1 in breast cancers than normal tissues and found that patients with overexpression of TopBP1 in the tumors have significantly shorter overall survival time and shorter progression free survival time than those without overexpression of TopBP1. Therefore, TopBP1 may be an important prognostic marker for aggressive subgroups of breast cancer [37]. Since TopBP1 is involved in regulation of promoter binding activity of p53 during normal growth, the authors suggest that, its abnormally high level may potentially inactivate p53 and contribute to an aggressive behavior of breast tumors [37].

Going and coworkers [34] who analyzed the TopBP1 expression by immunohistochemistry in normal and cancerous breast tissues reported the significant changes in TopBP1 localization. In normal breast tissue, TopBP1 staining was almost entirely nuclear in location, although in rare cells some cytoplasmic staining was occasionally detected [34]. The nuclear localization is consistent with the function of TopBP1 which is involved in DNA damage response and initiation of DNA replication [4, 15]. However, the pattern of TopBP1 staining in human breast tissues changed from predominantly nuclear in normal epithelium, to nuclear and cytoplasmic or purely cytoplasmic in most of cancers [34]. Expression of TopBP1 was also reported in feline and canine mammary neoplasia [35, 36]. TopBP1 staining was predominantly nuclear, but in some tumors there was additional cytoplasmic staining [35, 36]. Expression of TopBP1 protein in feline and canine mammary neoplasia was positively correlated with histological grade and additionally to nuclear, cytoplasmic staining was observed as the degree of malignancy increased [34–36]. Our results concerning localization of TopBP1 are consistent with results ascribed as above*.* We also found in normal tissue mainly nuclear localization of TopBP1 whereas in most cancer tissue samples TopBP1 localized also in cytoplasmic compartment. The percentage of samples with cytoplasmic expression of TopBP1 increased with increasing histological grade. However, there was no significant association between level and intracellular localization of TopBP1 protein in hereditary breast cancer and other clinicopathological parameters as estrogen and progesterone receptors status, appearance of metastasis in the axillary lymph nodes and type of cancer.

There are no studies that demonstrated expression of *TopBP1* at mRNA level and its relationship to TopBP1 protein level in normal and cancerous breast tissues. The results of our present study surprisingly showed lower level of *TopBP1* mRNA in cancer samples compared to normal tissues. We also found that expression of *TopBP1* mRNA contrary to TopBP1 protein level was inversely correlated with histological grade of hereditary breast cancers. Moreover, expression of *TopBP1* mRNA was significantly down-regulated in the lobular carcinoma compared to the ductal carcinoma. However, we did not find any correlation between TopBP1 protein level and type of breast cancer.

At present we do not know the reason of discrepancies between *TopBP1* mRNA and protein level. The lack of compatibility between alteration in mRNA and protein levels confirms that there is no simple and direct relation between transcriptom and proteome. Many factors may influence on the protein content in the cells. We suggest that in regulation of *TopBP1* mRNA and protein levels a kind of negative feedback control mechanism may be involved. Down-regulation of mRNA concurrent with up-regulation of protein level may occur when a protein half-life is increased due to stabilization because components involved in the protein’s normal turnover may be disrupted or the protein may be stabilized through protein–protein interactions. There is possibility that in cancer cells TopBP1 is more stable and can be accumulated. We do not know what could make TopBP1 more stable but it can be associated with TopBP1 mislocalization in cancer cells. Accumulated TopBP1 may act as a repressor of transcription. Our results showing inverse correlation between *TopBP1* mRNA and protein levels in breast cancers seem to confirm this hypothesis. Expression of *TopBP1* gene is regulated by E2F1 and Egr-1 [38, 39]. On the other hand TopBP1 regulates activity of E2F1 and high level of this protein suppresses E2F1 transcriptional activity without affecting E2F1 protein levels [8, 14]. Thus, decreased transcriptional activity of E2F1 by TopBP1 may repress the expression of E2F1 target genes, including *TopBP1*.

In summary, the association between *TopBP1* mRNA and protein expression and aggressive behavior of breast cancer could have a potential therapeutic implication. However, mechanisms of *TopBP1* expression regulation need to be elucidated.
